# Sarcopenia and Cognitive Function: Role of Myokines in Muscle Brain Cross-Talk

**DOI:** 10.3390/life11020173

**Published:** 2021-02-23

**Authors:** Lucia Scisciola, Rosaria Anna Fontanella, Surina, Vittoria Cataldo, Giuseppe Paolisso, Michelangela Barbieri

**Affiliations:** Department of Advanced Medical and Surgical Sciences, University of Campania "Luigi Vanvitelli", 80138 Naples, Italy; lucia.scisciola@unicampania.it (L.S.); rosariaanna.fontanella@unicampania.it (R.A.F.); surina@unicampania.it (S.); vittoria.cataldo@studenti.unicampania.it (V.C.); michelangela.barbieri@unicampania.it (M.B.)

**Keywords:** myokines, exerkines, skeletal muscle, sarcopenic, cognitive impairment

## Abstract

Sarcopenia is a geriatric syndrome characterized by the progressive degeneration of muscle mass and function, and it is associated with severe complications, which are falls, functional decline, frailty, and mortality. Sarcopenia is associated with cognitive impairment, defined as a decline in one or more cognitive domains as language, memory, reasoning, social cognition, planning, making decisions, and solving problems. Although the exact mechanism relating to sarcopenia and cognitive function has not yet been defined, several studies have shown that skeletal muscle produces and secrete molecules, called myokines, that regulate brain functions, including mood, learning, locomotor activity, and neuronal injury protection, showing the existence of muscle-brain cross-talk. Moreover, studies conducted on physical exercise supported the existence of muscle-brain cross-talk, showing how physical activity, changing myokines' circulating levels, exerts beneficial effects on the brain. The review mainly focuses on describing the role of myokines on brain function and their involvement in cognitive impairment in sarcopenia.

## 1. Sarcopenia

Sarcopenia is a common condition in older individuals, characterized by the progressive degeneration of muscle mass and function. It is associated with severe complications, which are falls, functional decline, frailty, and mortality [1]. The prevalence of sarcopenia varies from 9.9% to 40.4%, depending on its definition [2]. Nowadays, there is no consensus on defining the cut-off points, making sarcopenia diagnosis challenging. 

The pathogenesis of sarcopenia remains still poor clear and involves an interplay between sedentary lifestyle, aging, obesity, inflammation, and oxidative stress that affect muscle mass and function [3]. 

A sedentary lifestyle, defined as activities that do not increase energy expenditure, impacts muscle mass and metabolism. Indeed, only seven days of decubitus resulted in a loss of muscle mass and a prolonged period, 90–120 days, reduced 30% of the muscle volume [4,5]. Studies conducted on old immobilized animals have examined the effects of bed rest on skeletal muscle metabolism, demonstrating a disruption in the balance between protein synthesis and degradation in favor of catabolism [4,5]. 

Interestingly, aging alters both the homeostasis of skeletal muscle, compromising the equilibrium between cell regeneration and differentiation [6], and the rate of protein synthesis and degradation [7]. It is associated with reducing skeletal muscle stem cells (satellite cells) in type II fiber. Major pathways associated with changes in satellite cells during aging include Notch and Wnt signaling; the first one is associated with proliferation while the second with differentiation of muscle cells [8]. Studies demonstrated that the expression of Notch signaling decreased with age during aging [9], and Wnt canonical pathway switched to not canonical pathway resulting in the inability of satellite cells to self-renewal [10]. However, the hypothesis that loss of satellite cell activity is the cause of sarcopenia has been confuted. In male sedentary mice, the depletion of satellite cells, resulting in impaired muscle regeneration, did not contribute to muscle size or fiber type composition, despite low regenerative capacity, but contributed to age-related muscle fibrosis [11].

With advancing age, the intake of amino acids is inadequate, resulting in a decreased protein synthesis rate and the proteolysis system's inability (ubiquitination and lysosomal degradation) to remove oxidized proteins, inducing a progressive decline in skeletal muscle mass and function [7,12]. 

Pathogenic inter-relationship between adipose tissue and muscle is also crucial in sarcopenia and contributes to functional and physiological impairment. Obesity is characterized by increased production of fatty acids (FAs) that are not only stored in adipose tissue (AT) but can outflow and accumulate ectopically in skeletal muscle [13]. FAs, in the form of triglycerides (TG), diacylglycerols (DAG), and ceramides, accumulate both in intermuscular adipose tissue (IMAT) as in intramyocellular lipids (IMCLs), inducing impaired single-fiber contractility via mitochondrial dysfunction, impaired β-oxidation of FAs, and increased reactive oxygen species (ROS) production, leading to lipotoxicity and insulin resistance (IR) [14,15].

These events' primary outcome is muscle fiber insufficiency with a decline in muscle mass and function [16]. Indeed, IMCLs attract immune cells, such as M1-type macrophages, mast cells, Th1, Th17, and other cells, that produce an array of pro-inflammatory cytokines [17,18,19,20]. Activated adipocytes produce pro-inflammatory adipokines, like leptin, osteopontin, chemerin, and a lower expression of SIRT1 in the subcutaneous abdominal fat [21], creating a pro-inflammatory vicious circle providing local and systemic, chronic low-grade inflammation [22,23], which is also related to glucose metabolism derangement [24]. Furthermore, this unfavorable adipokines/cytokine profile increases IR and contributes to ectopic fat distribution [25]. 

Besides, mitochondria oxidative capacity and NAD^+^ biosynthesis are reduced in sarcopenic muscles. A study conducted on 119 sarcopenic individuals demonstrated that PGC-1α/ERRα signaling, oxidative phosphorylation, and mitochondrial proteostasis genes are downregulated. These changes decreased mitochondria, mitochondrial respiratory complex expression and activity, and NAD^+^ levels via perturbed NAD^+^ biosynthesis [26].

Moreover, AT inflammation and skeletal muscle functionality are exacerbated by senescence-associated secretory phenotype (SASP) produced by senescent cells [27,28]. Studies suggested that senescent cells accumulate in skeletal muscle of aged rodents and elderly people demonstrating the expression of p16Ink4a and positive results of the senescence-associated β-galactosidase assay [29].

## 2. Sarcopenia as a Risk Factor for Cognitive Decline

In the literature, it is well documented that sarcopenia increases the risk of cognitive decline [30]. Despite the contradictory results that could be due to different criteria and cut-off points to assess used sarcopenia components [31], a recent systemic review and meta-analysis demonstrated that the association between sarcopenia and cognitive impairment was independent of the study population, sarcopenia definition, and cognitive impairment degree (odds ratio 2.2, 95% CI 1.2–4.2) [32].

In particular, a cross-sectional study based on 3025 women aged 75 years and older demonstrated an association between muscle strength, a central component of sarcopenia, and cognitive function. Lower handgrip (HGS), used to measure muscle strength, was associated with cognitive impairment, measured by a short portable mental status questionnaire (SPMSQ) (OR 1.81 and 95% confidence interval: 1.33–2.46) [33,34]. Which cognitive domains are affected by muscle strength are poorly described. A cross-sectional study, conducted on 1799 participants aged more than 60 years old, demonstrated a higher digit symbol substitution test (DSST) score, used to measure visuospatial and motor speed was more significant in higher quadriceps strength groups indicating that muscle strength was associated with frontal lobe executive functions [34]. Another study of 555 participants, all aged 85 years at baseline, suggested that HGS was associated with processing speed and memory function [35]. 

Even muscle mass is considered a predictor of cognitive decline, the link between muscle mass and cognitive impairment is not consistently documented [31].

Although the exact mechanisms involved have not yet been defined, risk factors may partially explain the association between cognitive decline and sarcopenia. Direct cross-talk between muscle and brain, mediated by exercise-induced myokines release, has been demonstrated [36,37]. Physical activity restores and maintains cognitive functions and metabolism [38,39] and ameliorates the process of neurological diseases [40], inducing muscle cells, metabolically active, to produce and release myokines. It was proposed that all factors released in response to exercise should be termed "exerkines" [41].

## 3. Role of Physical Exercise in Muscle and Brain Cross-Talk

Physical activity is a non-pharmacological intervention that ameliorates brain function [42]. It has been reported that exercise increases the volume and intensifies the prefrontal cortex's function, hippocampus, which are neuronal regions related to memory and cognition [43,44,45,46]. Studies conducted on people with AD, the most common form of dementia, have demonstrated that exercise can improve cognitive and physical function [47]. Moreover, activity was associated with a 30–40% reduction in the risk of developing AD than physically inactive individuals [48]. 

A longitudinal observational study demonstrated an association between physical activity and a lower likelihood of cognitive decline (RR 0.65, 95% CI 0.55–0.76) [49]. Similar results were obtained from another study that demonstrated that the group with cognitive impairment had more deficient performance gait speed test than the control group [50]. The exercise-induced improvement in cognitive function was also demonstrated in older adults. A meta-analytic study examined aerobic fitness effects on cognitive vitality of healthy but sedentary older adults. The study has indicated that physical activity impacts positively on cognition [51]. 

Physical exercise mediates the beneficial effects promoting cerebral angiogenesis, increasing neurogenesis and plasticity of the hippocampus, increasing cerebral blood flow, diminishing blood-brain barrier (BBB) permeability and function [52], and enhancing oxygen-rich blood delivery to the brain [53,54,55,56].

In skeletal muscle, physical exercise activates compensatory and adaptive mechanisms to obtain energy that can be reached via metabolic regulation or changes in gene expression [57]. Exercise regulates myokines' expression, contributing to autocrine regulation of metabolism in the muscle and paracrine/endocrine regulation of other adjacent/remote organs [42]. Studies conducted on exercise showed that physical activity, increasing circulating levels of myokines in the bloodstream, exert beneficial effects on the brain. The myokines regulate brain functions, including mood, learning, locomotor activity, and protecting neuronal injury in animal or in vitro models [41,42,55]. 

**Figure 1:**
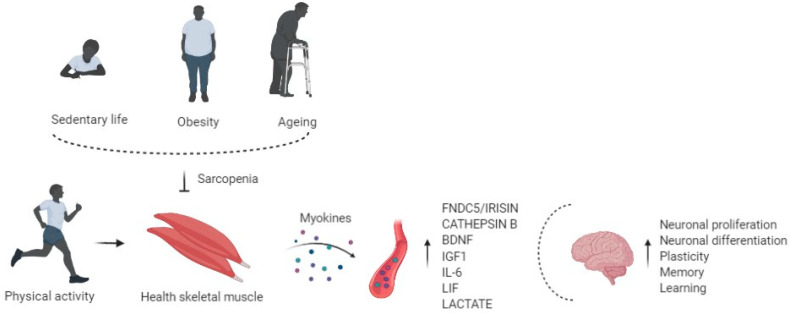
Physical activity enhances circulating levels of myokines in the bloodstream, affects the brain regulating neuronal proliferation and differentiation, plasticity, memory, and learning. Risk factors of sarcopenia, such as physical inactivity, obesity, and aging, alter the myokines' production and release, impairing cognitive function.

So, altered synthesis and production of myokines due to physical inactivity may be associated with adverse implications in the brain, such as cognitive impairment and neurogenerative events [58], showing that muscle may influence the health of the brain (Figure 1).

## 4. Exercise Induced Myokines and Brain Function

Myokines have provided a new paradigm and a conceptual basis for understanding the cross-talk between muscle and other organs or tissues.

The skeletal muscle was identified as an endocrine organ with a high capacity to produce, express, and secrete various factors, which are classified as myokines [59,60].

Myokines are cytokines and other peptides produced following skeletal muscle contractions and exert autocrine, paracrine, and endocrine effects [61].

Recent research identified over 600 myokines [62]; however, their specific bioactivity remains largely undescribed and poorly understood [63].

Myokines are involved in muscle proliferation, differentiation, and regeneration [64,65], but also mediate signaling between muscle and liver, gut pancreas, adipose tissue, bone, brain, vascular bed, skin, and present anticancer effects [61,66,67,68]. Emerging evidence indicates that myokines improve human health and ameliorate multiple diseases [69,70,71]. Indeed, myokines regulate systemic glucose homeostasis, lipid metabolism, enhance insulin sensitivity, and induce white adipose tissue (WAT) browning [72,73,74]. 

Myokine signaling mediates the muscle-brain endocrine loop, promoting relationship building between muscle and brain (Table 1) [36,37]. 

### 4.1. FNDC5/Irisin

Fibronectin type III domains containing protein 5 (FNDC5) is a glycosylated type I membrane protein [75]. Following proteolytic cleavage of FDN5C, irisin is generated as a peptide of 112 amino acids (aa 29–140), and it is released into the circulation [76]. 

FNDC5/IRISIN is described as an exercise-induced myokine; indeed, skeletal muscle produces the most quantity of irisin's total circulating levels. In skeletal muscle exercise, inducing the activation of peroxisome proliferator-activated receptor gamma coactivator 1-alpha (PGC-1α), a key regulator of skeletal muscle plasticity after exercise promotes the synthesis and secretion of irisin [77]. Several studies demonstrated that physical activity in mice and humans increases Fndc5 mRNA in skeletal muscle cells [78,79,80]. 

Irisin circulating concentration was significantly higher in individuals after endurance exercise [81].

In the hippocampus, a region involved in memory and spatial awareness, exercise leads Fndc5 expression in a PGC-1α-dependent manner in mice model. Pgc1α −/− mice did not present FNDC5 expression [78].

Also, irisin stimulates neuronal proliferation and differentiation and contributes to the exercise neuroprotective effects through activation of protein kinase B (PKB) and extracellular signal-regulated kinases 1/2 (ERK1/2) signaling pathway [82].

Recently, research suggested the role of irisin in regulating synaptic function and memory in mouse models of AD [83]. In the brain of Fndc5 −/− mice, a mice mole of AD, synaptic plasticity and long-term potentiation are compromised, while the FNDC5/irisin re-expression rescued synaptic plasticity and memory impairment [83]. 

Moreover, irisin increase brain-derived neurotrophic factor (BDNF) expression in the brain, which is involved in the cognitive function [41].

### 4.2. Cathepsin B

Cathepsin B (CTSB) belongs to a family of lysosomal cysteine proteases [84].

Exercise induces the Ctsb gene expression in muscle, which promotes BDNF synthesis in hippocampal stimulating neurogenesis in mice model [85]. These findings in rodents are supported by results obtained in rhesus macaque and in human, where 4 months of treadmill exercise elevated CTSB level in plasma [85]. Crossing BBB, skeletal muscle-derived CTSB induces hippocampal upregulation of BDNF and doublecortin, regulating synaptic plasticity, cell survival, and neuronal migration [86].

Another study showed that mice treated for 1 week with AICAR, which stimulates AMP-activated protein kinase (AMPK), presented an improvement of hippocampal neurogenesis and cognitive function [87]. These findings revealed that endurance exercise, activating AMPK in skeletal muscles, induced a release of CTSB, which may be associated with exercise-induced improvement of cognitive function, such as neurogenesis, memory, and learning [41,87,88]. Cathepsin B knock-out mice showed reduced adult hippocampal neurogenesis and impaired spatial learning and memory. Stimulation of adult neuro-progenitor cells with recombinant cathepsin B increased neurogenesis [85].

### 4.3. BDNF

Brain-derived neurotrophic factor (BDNF) belongs to the neurotrophin family, and it is a crucial mediator of beneficial effects of exercise on the brain [86]. In response to acute exercise and exercise training, BDNF is abundantly expressed in the brain, but several studies verify its expression also in skeletal muscle [89]. It is not well understood if muscle-derived BDNF is released in the bloodstream and mediates muscle-brain cross-talk. Instead, more evidence suggests that FNDC5 and Cathepsin B, crossing the BBB, positively induce BDNF expression in the hippocampus via PKB activation and “cAMP response element-binding protein” (CREB) signaling inducing an increment of BDNF [90]. BDNF influences cognitive function activating (Phosphoinositide phospholipase -γ) PLC- γ, Phosphoinositide 3-kinase (PI3K), and ERK pathways that together affect synaptic plasticity [91]. 

BDNF, increasing the growth and proliferation of hippocampal dentate gyrus cells, is involved in neuronal differentiation, plasticity, cell survival, hippocampal function, showing a dominant role in mediating the effects of physical activity on cognitive changes [86]. 

Exercise-induced BDNF was shown to decrease the production of toxic amyloid β peptides, which could be important in treating Alzheimer’s disease (AD). Patients with neurodegenerative diseases, like AD, Parkinson's disease, and depression, presented low serum levels of BDNF [92]. 

### 4.4. IGF1

Insulin-like growth factor1 (IGF-1) is an essential factor in brain neurogenesis and cognitive function; therefore, IGF-1 signaling may play a key role in muscle-brain cross-talk [37,93]. A primary source of IGF-1 is the liver; however, it is produced by skeletal muscle during physical activity [94]. Aerobic exercise not only increases the neuronal uptake of IGF-1 but stimulates IGF-1 signaling pathways inducing PKB-CREB-mediated BDNF expression, followed by neurogenesis and neuron survival [95].

Studies have demonstrated that, in older adults, aerobic exercise, increasing IGF-1 and consequently BDNF, significantly increased hippocampal volume and connectivity [96].

IGF-1 is involved in several brain functions, including neurotrophic, angiogenic, and metabolic proprieties [42].

### 4.5. IL-6

Interleukin 6 (IL-6) was the first myokine found to be secreted into circulation in a tumor necrosis factor (TNF)-independent manner [97] in response to muscle contractions, with a considerable increase in plasma up to 100-fold in response to exercise [98]. During exercise, circulating IL-6 levels increased without any sign of muscle damage [99]. Physical activity increased the expression of IL-6 mRNA and protein levels in the brain [100,101]. Two weeks of voluntary wheel running increased IL-6 expression in the hippocampus in mice, resulting in downregulation of pro-inflammatory cytokines and inflammation, suggesting that IL-6 may protect the brain, reducing harmful inflammatory responses [102]. 

In vitro studies showed that IL-6 promotes the survival and differentiation of neural cells [103,104], protecting against Ca2+ and ROS excitotoxicity [105] Il-6 plays a role in neurodegenerative diseases such as AD. In vitro studies demonstrated that the addition of Il-6 in a culture medium increased neurotoxicity caused by Aβ in cortical neurons. However, in vivo, IL-6 increased astrocytes and microglia cells’ activation, improving plaque Aβ clearance, showing its neuroprotective properties [106]. 

Further investigations should be carried out to better identify the role and mechanisms of myokine IL-6 in cognitive function.

### 4.6. LIF

Leukemia inhibitory factor (LIF) belongs to the IL-6 family and it is produced by different organs or tissues, including cardiac muscle, neuronal tissue, and skeletal muscle [107]. LIF is involved in the astrocyte's development, oligodendrocytes survival, and recovery processes after injuring the spinal cord in mice [42,108]. LIF is an exercise-induced myokine and via autocrine or paracrine signaling induces hypertrophy and regeneration of skeletal muscle [110,111,112]. It was also demonstrated that LIF, via PKB/extracellular signal-regulated-mediated c-fos induction, protects against amyloid β-induced neurotoxicity [112].

### 4.7. L-Lactate

L-Lactate is a metabolite from contracting skeletal muscle [113]. L-Lactate is released from muscles during short-term exercise, even from muscles at rest, during recovery from short-term exercise, and long-lasting exercise [114].

Extended findings conclude that L-Lactate is a component of the "exercise pill." L-Lactate is a hormone involved in memory and neuroprotection, and it seems to be an essential substrate for neuronal metabolism and long-term potentiation (LTP) maintenance [115]. L-Lactate produced in exercised mice crossed the blood-brain barrier to induce expression of Bdnf and signaling of the BDNF receptor tropomyosin receptor kinase B (TrkB) in the hippocampus, resulting in the promotion of learning and memory formation [116].

Acute and high-intensity exercise lactate promotes angiogenesis in the brain by binding with hydroxycarboxylic acid receptor 1 (HCAR1) [42]. L-lactate's daily injection, simulating blood lactate levels observed during exercise, increased vascular endothelial growth factor (VEGF) levels, and, consequently, microvascular density in the dentate gyrus [117]. 

L-Lactate potentiates N-methyl-D-aspartate receptor (NMDA) glutamate receptor-mediated signaling, playing a central role in neuronal plasticity and memory processes; L-Lactate activates a cascade of molecular events that end up with stimulation of the expression of synaptic plasticity-related genes, such as activity regulated cytoskeleton associated protein (Arc), c-Fos, and Zif268 [118].

## 5. Conclusions

The remarkable improvements in life expectancy resulted in a longer lifespan that brings new opportunities for elder people. However, these opportunities and contributions depend on one factor that is health. 

Indeed, aging-chronic diseases negatively impact health and consequently affect the quality of life and represent major public health concerns. In the last decade, strong emphasis was given to sarcopenia, defined as age-related loss of muscle mass and function [6]. 

Epidemiological evidence suggests that sarcopenia is associated with accelerated cognitive changes that lead to cognitive impairment [30]. The exact mechanism relating to sarcopenia and cognitive impairment is not well elucidated, but the existence of muscle-brain endocrine loop mediated by myokines is demonstrated [31]. Myokines are muscle-produced factors that improve brain function as cognition, memory, and motor coordination [36]. Sarcopenia is linked to a reduction in the regenerative capacity of skeletal muscle stem cells and an altered rate of cellular regenerative and differentiation; therefore, this could result in a compromised production and secretion of myokines with a negative consequence on brain function [1]. On the contrary, exercise regulates myokines' expression contributing to autocrine regulation of metabolism in the muscle and to paracrine/endocrine regulation of other adjacent/remote organs [37].

Even though specific bioactivity remains undescribed and poorly understood [59], clarifying better how myokines are secreted and released from muscle fiber, their effect on the brain will help understand the link between muscle, exercise, and brain function. It may also be possible to develop small compounds derived from myokines to provide treatment of neurodegenerative diseases.

## Figures and Tables

**Table 1:** Mechanisms of action and effects of myokines on the brain.

Myokine	Effects on the Brain	Mechanisms of Action
**FNDC5/Irisin**	Neuronal proliferation and differentiation, synaptic function, memory [75,76,77,78,79,80,81,82,83]	PKB and ERK1/2 signaling pathway [82]
**Cathepsin B**	Neurogenesis, memory, Learning [84,85,86,87]	BDNF synthesis [86,87]
**BDNF**	Synaptic plasticity, neuronal differentiation, cell survival, hippocampal function [89,90,91,92]	PI3K and ERK signaling pathway [91]
**IGF1**	Neurogenesis and neuron survival, neurotrophic, angiogenic, and metabolic proprieties [93,94,95,96]	BDNF synthesis [96]
**IL-6**	Survival and differentiation Further investigation are needed [97,98,99,100,101,102,103,104,105,106]	To be investigated
**LIF**	Astrocyte’s development, oligodendrocytes survival amyloid β-induced neurotoxicity [107,108,109,110,111,112]	AKT/extracellular signal-regulated-mediated c-fos induction [112]
**L- Lactate**	Memory, learning, neuroprotection, neuronal plasticity, neuronal metabolism, LTP maintenance, Angiogenesis [113,114,115,116,117,118]	BDNF synthesis; Hydroxycarboxylic acid receptor 1 (HCAR1); VEGF synthesis; NMDA glutamate receptor-mediated signaling; Arc, c-Fos, and Zif268 synthesis [113,114,115,116,117,118]

## Data Availability

Not applicable.
